# Diagnosis and Medical Treatment of Acute and Chronic Idiopathic Pouchitis in Inflammatory Bowel Disease

**DOI:** 10.3390/medicina60060979

**Published:** 2024-06-13

**Authors:** Corina Meianu, Tudor Stroie, Doina Istratescu, Carmen Monica Preda, Mihai Mircea Diculescu

**Affiliations:** 1 Gastroenterology Department, Fundeni Clinical Institute, 022328 Bucharest, Romania; 2 Faculty of Medicine, “Carol Davila” University of Medicine and Pharmacy, 050474 Bucharest, Romania

**Keywords:** pouch, ulcerative colitis, ileal pouch-anal anastomosis, biologics, small molecules

## Abstract

Despite the decreased rates in inflammatory bowel disease (IBD) colectomies due to high advances in therapeutic options, a significant number of patients still require proctocolectomy with ileal pouch-anal anastomosis (IPPA) for ulcerative colitis (UC). Pouchitis is the most common complication in these patients, where up to 60% develop one episode of pouchitis in the first two years after UC surgery with IPAA with severe negative impact on their quality of life. Acute cases usually respond well to antibiotics, but 15% of patients will still develop a refractory disease that requires the initiation of advanced immunosuppressive therapies. For chronic idiopathic pouchitis, current recommendations suggest using the same therapeutic options as for IBD in terms of biologics and small molecules. However, the available data are limited regarding the effectiveness of different biologics or small molecules for the management of this condition, and all evidences arise from case series and small studies. Vedolizumab is the only biologic agent that has received approval for the treatment of adult patients with moderately to severely active chronic refractory pouchitis. Despite the fact that IBD treatment is rapidly evolving with the development of novel molecules, the presence of pouchitis represents an exclusion criterion in these trials. Recommendations for the approach of these conditions range from low to very low certainty of evidence, resulting from small randomized controlled trials and case series studies. The current review focuses on the therapeutic management of idiopathic pouchitis.

## 1. Introduction

The rates of colectomy for severe refractory ulcerative colitis (UC) have decreased over the past three decades due to great advances in the field of inflammatory bowel disease (IBD) treatments and the availability of new molecules. However, colectomy is still needed for UC patients with a prevalence of around 10% at 10 years [1]. The preferred surgical option in these cases is restorative proctocolectomy through ileal pouch-anal anastomosis (IPAA), whereby the most common surgical reconstruction of the pouch is the J pouch. The surgical procedure consists of removing the colon and rectum and uses the terminal ileum to form an internal pouch that is further attached to the anal sphincter muscle in order to replace the removed rectum and to fulfill the function of a reservoir for the storage of stools and control of bowel movements. The J pouch is constructed using 30–40 cm of terminal ileum that is further folded into two segments of 15 to 20 cm. A stapled side-to-side anastomosis of the two segments is performed via enterotomy at the pouch apex. A blind loop is closed using a linear stapled and additional continuous sutures [2]. Restorative proctocolectomy aims to offer the patients comfort with their medical condition and an improved quality of life. Although it has been considered that colectomy is curative for UC and it ends the burden associated with IBD, numerous complications arise with the J pouch: non-inflammatory, mechanical related to surgery, functional or inflammatory. Of the inflammatory conditions, the most common is pouchitis with an increased prevalence of up to 60% of patients in the 2 years after IPAA [3,4,5]. Similar with UC, physiopathology of idiopathic pouchitis involves an inadequate immune response to local microbiota in an organism with genetic susceptibility [6]. In clinical practice, the treatment of pouchitis is challenging. Usually, acute pouchitis has a favorable response to antibiotics, but up to 15% of patients develop a chronic phenotype, either antibiotic-dependent or antibiotic refractory, which needs the initiation of immunosuppressive treatments. However, many cases have an inadequate response to biologics and small-molecule treatment that can lead to pouch failure and require pouch excision [7,8]. For chronic pouchitis, current recommendations suggest the use of the same advanced immunosuppressive agents as for the treatment of CD and UC. Available data are very limited in terms of the efficacy of different biologic agents or small molecules for the treatment of chronic pouchitis. Also, there is no positioning among these therapies in the first or second lines of treatment. An explanation for this lack of data could be that in most randomized controlled trials of these drugs, patients with pouch represent and exclusion criterion. Randomized controlled trials on drug efficacy and safety that will include patients with pouch or that will focus mainly on this condition are needed. This will further lead to consensus guidelines and to a better management of these conditions. In this review, we discuss about the diagnosis and classification of pouchitis, with a focus on therapeutic agents in the treatment of idiopathic pouchitis, including advanced molecules. The current review focuses on the therapeutic management of idiopathic pouchitis, including novel biologics and small molecules, and discusses the potential benefit of cellular therapies and mesenchymal-cell therapies as future treatment perspectives.

## 2. Materials and Methods

To collect evidence for this paper, we performed a PubMed database search for studies published between 1 January 2000 and 30 April 2024. Key search terms were as follows: “restorative proctocolectomy”, “pouch”, “pouchitis”, “ileal pouch-anal anastomosis”, “ulcerative colitis”, “antibiotics”, “probiotics”, ”corticotherapy”, “immunomodulators”, “biologics”, “small molecules”, “infliximab, “adalimumab”, “ustekinumab”, “vedolizumab”, “tofacitinib” and “upadacitinib”. Abstracts of the articles that discussed pouchitis, definitions, classification, pathogenesis and therapeutic management of this condition were reviewed and relevant articles were used for citation.

## 3. Classification of Pouchitis, Causes and Pathogenesis

Pouchitis is classified into either idiopathic (primary) or secondary, according to the cause.

The etiology of primary or idiopathic pouchitis is not clearly defined but it is thought that dysbiosis plays an important role. Pouch construction determines local fecal stasis that leads to colonic metaplasia in the initial small bowel mucosa of the pouch that promotes an abnormal immune response in genetically predisposed organisms, which leads to inflammation; a similar mechanism that it is seen in UC [9,10,11]. Biopsies from normal pouch mucosa suggested a transition from common ileal bacterial species to a colon-like microbiota with high expression of *Proteobacteria* spp. and a decrease in *Bacteroidetes*, *Firmicutes*, *Lachnospiraceae* and *Ruminococcaceae* species. A decrease in the diversity of the bacterial population was also documented in patients with pouchitis compared to those with normal pouch [12,13]. Innate and adaptive immune response has been shown to be abnormal in patients with or without pouchitis. Increased epithelial expression of the “pore forming” tight junction proteins claudin 2 and dendritic cells were identified in biopsy specimens from pouch mucosa after restorative proctocolectomy for UC [14]. The expression of Toll-like receptors (TLRs) is also altered, with a decreased expression of TLR3 and increased expression of TLR5 in normal ileal pouch, and an upregulation of TLR2 expression in pouchitis and upregulation of TLR4 in both normal pouch and pouchitis [15,16,17]. An increased cytokine production of IL-1 beta, IL-6, IL-8 and TNF-alpha was seen in pouchitis [18,19]. Risk factors that were identified to be associated with pouchitis include the following: extensive UC and backwash ileitis before colectomy; the presence of extraintestinal manifestations; non-smoker status; IL-1 receptor antagonist gene polymorphism; and the presence of pANCA (antineutrophil cytoplasmic antibodies) [20,21,22,23]. Secondary pouchitis is determined by identifiable factors such as infections (e.g., *Clostridium difficile* or cytomegalovirus), NSAID-associated pouchitis, ischemic pouchitis and radiation pouchitis, or induced by fecal stasis in the case of pouch outlet obstruction or pelvic floor dyssynergia [24,25,26]. Another possible cause of secondary pouchitis is the immune-mediated phenotype, which is associated with an inflammation of the pouch and of the pre-pouch ileum, positive autoantibodies, high serum levels of immunoglobulin (Ig) G4, an increase in Ig G4-expressing cells in the mucosa of the pouch and the association with other autoimmune conditions. The treatment consists of controlling the concurrent autoimmune disorders and often requires immunosuppressive agents [27]. Pouchitis associated with primary sclerosing cholangitis (PSC) is another entity of immune-mediated pouchitis. About 70% of patients with PSC have underlying IBD, with a stronger association with UC than with CD at an estimated rate of 75% of cases. UC associated with PSC seems to have a distinct phenotype: usually, it presents itself with a mild activity, rectal sparing and ileal involvement known as “backwash ileitis” [28]. The risk of developing pouchitis is increased in patients with primary sclerosing cholangitis. Evidence suggests that the cumulative risk of pouchitis at one, two, five and ten years is 15.5%, 22.5%, 36% and 45.5% in patients without PSC and 22%, 43%, 61% and 79% in patients with concomitant PSC. PSC-associated pouchitis has a high risk of progression to chronic pouchitis in 68.1% of patients, and in clinical practice, it represents a treatment challenge associated with antibiotic resistance. The etiology of PSC-associated pouchitis remains unknown. One possible physiopathological mechanism may involve pouch dysbiosis. Patients with PSC have an increase in *Escherichia*, *Fusobacterium*, *Ruminococcus* and *Megaspera genera*, with a reduction in *Prevotella*, *Blautia* and *Bacteroides* when compared with patients without PSC. Also, the concentration of secondary bile acids in stool has been found to be inversely proportional with IBD activity, but studies regarding this mechanism are lacking in patients with restorative proctocolectomy and IPAA [29,30]. Treatment for primary sclerosing cholangitis-induced pouchitis includes budesonide, oral vancomycin and vedolizumab [29,31]. According to clinical studies, pouchitis is classified into either acute or chronic, taking into consideration the duration of symptoms. Symptoms that last more than 4 weeks define chronic pouchitis. Recurrent pouchitis with more than 3–4 episodes/year can also be considered chronic pouchitis. Considering the response to antibiotic therapy, pouchitis is classified into antibiotic responsive, antibiotic-dependent and chronic antibiotic-refractory pouchitis (CARP). Antibiotic responsive pouchitis defines pouchitis that has a complete resolution of symptoms when treated with antibiotics; antibiotic-refractory refers to the form of disease that does not improve clinically after a 2–4 week course of conventional antibiotics; and antibiotic-dependent disease refers to the pouchitis that responds well to antibiotics but relapses shortly within 4 weeks after stopping antibiotics or has a relapsing course of more than 3–4 episodes per year [32].

## 4. Diagnosis of Pouchitis

Normal intestinal transit in patients with IPAA is considered to be up to 4–8 stool emissions during the day and 1–2 emissions at night [33,34]. Pouchitis is diagnosed based on symptoms and endoscopic and histologic findings. Clinical symptoms associated with pouchitis are urgency, increase in stool frequency, decrease in stool consistency and lower quadrant abdominal pain [35]. There are no validated scoring systems for the diagnosis and assessment of pouchitis. Although not validated, the Pouchitis Disease Activity Index (PDAI) was formulated (Table 1). It takes into account clinical symptoms, endoscopic aspect and acute histologic findings with a score of at least seven defining pouchitis [36]. The Heidelberg Pouchitis Activity Score (PAS) takes into account the histological aspect of chronic inflammation and describes three grades of pouch inflammation [35]. It is important to note that symptoms, endoscopic findings and histology are not well correlated to each other, so in the case of the clinical suspicion of pouchitis, an endoscopic evaluation with biopsies of the lesions needs to be performed to assess pouch inflammation in order to distinguish between pouchitis and other pouch disorders such as functional disorders of the pouch. Endoscopic findings in pouchitis consist of the presence of decreased vascular pattern, friability of the mucosa, granularity, erythema, erosions and ulcerations [37]. Serological markers such as C-reactive protein (CRP) and fecal calprotectin can be further used to assess inflammation in disease monitoring [38].

## 5. Treatment of Idiopathic Pouchitis

### 5.1. Probiotics

Several probiotic formulations have been studied for the primary prevention and for the treatment of recurrent pouchitis. The De Simone formulation, a mixture of eight different strains of Lactobacillus, Bifidobacterium and Streptococcus thermophilus, has been tested for the prevention of pouchitis with positive results. A placebo-controlled, randomized and double-blinded study that included 40 patients with restorative proctocolectomy and IPAA showed at one-year follow-up that 10% of patients who administered De Simone formulation had an episode of acute pouchitis compared to 40% of patients in the placebo group [39]. Also, a Cochrane systematic review showed the benefit of the De Simone formulation to placebo in preventing pouchitis [40]. Another double-blinded placebo-controlled randomized trial showed a high efficacy of the De Simone formulation in preventing pouchitis episodes in patients with chronic pouchitis [41]. Bifidobacterium longum was also studied in a randomized controlled study with favorable outcome for the prevention of pouchitis [40]. Another tested formulation, Clostridium butyricum MIYAIRI, showed significantly lower rates of pouchitis episodes at 2 years in treated patients [42]. The American Gastroenterological Association (AGA) recommends using probiotics in patients with recurrent episodes of pouchitis that respond to antibiotics for maintenance of remission but suggests avoiding them in patients with infrequent episodes due to the burden associated with long-term excessive use of probiotics [43]. The European Crohn’s and Colitis Organization (ECCO) suggests the maintenance of antibiotic-induced remission in chronic pouchitis with the De Simone formulation [44].

### 5.2. Antibiotics

There is one randomized-controlled trial that evaluated the efficacy of tinidazole as a preventive therapeutic agent for pouchitis. The study included 38 patients with IPAA after proctocolectomy for UC who were randomized 2:1 to receive either tinidazole 500 mg or placebo for 1 year. At the end of the follow-up period, only 8% of the patients in the tinidazole group developed pouchitis compared to 38.5% of patients in the placebo group [45]. Taking into consideration the lack of consistent data and the adverse effects associated with long-term use, antibiotics are not recommended for primary prophylaxis in pouchitis [46,47]. There are few placebo-controlled trials that evaluated the efficacy of metronidazole and ciprofloxacin for the treatment of pouchitis. Two randomized trials compared the efficacy of metronidazole versus ciprofloxacin. In one trial, sixteen patients with pouchitis were randomized to receive either ciprofloxacin 1 g/day or metronidazole 20 mg/kg/day for 2 weeks. Both drugs determined a significant reduction in PDAI score with a significantly greater reduction in the ciprofloxacin group. In the other trial, 52 patients with pouchitis were treated with metronidazole for 7 days, and 11 patients who had inadequate response to metronidazole were successfully treated with ciprofloxacin [48,49]. A double-blinded placebo-controlled trial involving 400 mg of metronidazole administered three times a day for 7 days that included 13 patients showed significant improvement in clinical symptoms, endoscopy and histology in patients treated with metronidazole versus placebo [50]. The combination of ciprofloxacin and metronidazole was evaluated in a four-week open-label trial in 44 patients with recurrent pouchitis and CARP. At the end of the treatment period, thirty-six (84%) patients entered in remission, which was defined as a combination of PDAI clinical score ≤ 2, endoscopic score ≤ 1 and a total PDAI ≤ 4. The study also assessed the quality of life with the Inflammatory Bowel Disease Questionnaire (IBDQ), which was well correlated with the PDAI score [51]. Rifaximin was studied in a placebo-controlled pilot study involving eight patients with pouchitis with higher rates of clinical remission at week four than the placebo group, although the difference was not statistically significant [52]. The combination of ciprofloxacin and metronidazole was assessed in two randomized controlled trials on eighteen and eight patients with chronic pouchitis in a two-week treatment period. Sixteen out of eighteen patients and seven out of eight patients, respectively, improved at the end of the treatment [53,54]. Long-term vancomycin in a dose of 125 mg twice daily was studied for the treatment of chronic pouchitis. A total of 51% of the patients had a clinical response at 4 weeks and 76% had a sustained response at three and six months [55]. Taking into consideration the limited data on other antibiotics, ciprofloxacin and metronidazole remain the preferred agents for the treatment of pouchitis with the most available evidence. Patients with acute pouchitis can be treated with a two-to-four-week course of ciprofloxacin 500 mg twice daily or metronidazole 20 mg/kg/day as a second line therapy. In the case of CARP, a combination of ciprofloxacin 1 g daily and metronidazole 1 g daily for 4 weeks can be used, or rifaximin 2 g daily, or metronidazole 1 g daily for 4 weeks [43,44]. In chronic antibiotic-dependent pouchitis, AGA recommends treatment with antibiotics with the lowest effective dose that can be used in an intermittent course, for example, one week per month or switching between ciprofloxacin, metronidazole and vancomycin every 1–2 weeks [43].

### 5.3. Corticotherapy

Corticotherapy can be used to induce remission in chronic antibiotic-refractory pouchitis. Twenty patients with active pouchitis who did not respond after a four-week antibiotic regimen were treated with budesonide controlled ileal release 9 mg/day for eight weeks in an open-label study. Fifteen of twenty patients achieved clinical remission according to PDAI and IBDQ [56]. Another small open-label study on five patients with chronic refractory pouchitis showed similar favorable results for budesonide 9 mg at eight weeks of treatment with remission obtained in four patients [57]. The efficacy of budesonide was tested in a retrospective trial in eighteen patients with PSC and pre-pouch ileitis and pouchitis at a dose of 9 mg for 1–3 months, followed by maintenance doses of 3–6 mg for 9 months. Although no significant change was observed in liver function tests, a significant improvement was seen in the endoscopic aspect of the pouch and of the pre-pouch ileum with regard to inflammation [58]. Other formulations of corticotherapy were also evaluated. A double-blinded randomized controlled trial investigated the benefit of budesonide enema compared to metronidazole for the treatment of pouchitis with similar positive outcomes at week four for both agents [59]. Oral beclomethasone dipropionate was tested in ten consecutive patients who did not respond to one-month antibiotic treatment for pouchitis. Eight out of ten patients achieved remission at week eight based on PDAI and IBDQ scores [60]. Corticotherapy seems to be effective in inducing clinical remission in patients with antibiotic-refractory pouchitis, with budesonide-controlled ileal release being the preferred formulation. However, corticotherapy should be avoided as a long-term therapy and salvage treatments should be considered [43,44].

### 5.4. Advanced Immunosuppressive Therapy

#### 5.4.1. Tumor Necrosis Factor-Alpha Antagonists (Anti-TNF-α)

Anti-TNF-α molecules have been evaluated for the treatment of chronic pouchitis as a rescue therapy. However, most studies use small numbers of patients and are retrospective. In a retrospective multicenter study of 33 patients treated with infliximab for chronic refractory pouchitis, 33% and 27% achieved clinical remission at week 26 and week 52 [61]. Adalimumab showed efficacy in eight patients with chronic pouchitis who were previously treated with infliximab, 13% achieving clinical remission and 62% achieving clinical response with adalimumab [62]. In a case series of ten patients with chronic pouchitis complicated with ileitis, treatment with infliximab led to clinical remission at week 10 in nine out of ten patients, eight of whom had sustained clinical remission through month 6 [63]. In a retrospective single-center study, 23 patients with CARP were treated with infliximab and 13 with adalimumab. Clinical remission was observed in 43.5% of patients in the infliximab group, and in 38.5% in the adalimumab group [64]. Infliximab treatment for CARP with or without pre-pouch ileitis in 25 patients obtained clinical response in 88% of patients at week 10 [65]. In a randomized, double-blinded, placebo-controlled study with adalimumab that included thirteen patients with CARP (wherein six patients received adalimumab and seven patients received placebo), the total PDAI improved in six patients in the adalimumab group compared to two patients in the placebo arm at week 12 [66]. Infliximab has proven high efficacy in IBD but it is also associated with high rates of loss of response. Long-term outcomes for infliximab therapy in chronic pouchitis were evaluated in 34 patients with CARP with or without pre-pouch ileitis. The study showed an overall infliximab failure in over half of the patients; 8% of patients had early failure and 44% of patients had secondary loss of response after a median follow-up period of 366 days [67]. In a case series, seven patients were treated with infliximab for CARP, with a complete clinical response based on PDAI score achieved at 1 year in five out of the seven patients [68]. A systematic review with meta-analysis that separately evaluated infliximab and adalimumab in 313 patients with pouchitis and Crohn’s disease (CD) complications of the pouch revealed that clinical remission at week 8 was obtained in 50% of patients and clinical remission at one year was obtained in 52% of patients [69]. A retrospective study from Spain evaluated the efficacy of infliximab, adalimumab and golimumab in first and secondary therapeutic lines in 145 patients with inflammatory conditions of the pouch, out of which 96 (66.2%) patients had chronic pouchitis. Clinical remission was achieved in 23.2% of patients in the infliximab group, 17.4% of patients in the adalimumab group and 14% of patients in the golimumab group in a follow-up period of a median of 25 months for infliximab and adalimumab and 15 months for golimumab [70].

#### 5.4.2. Anti-Integrin Therapy

Vedolizumab was evaluated for chronic pouchitis in a phase 4 double-blinded placebo-controlled study (EARNEST). Remission rates at week 14 assessed by PDAI were 31% (16 of 51 patients) in the vedolizumab group and 10% (5 of 51 patients) in the placebo group (*p* = 0.01). Rates of remission at week 34 favored vedolizumab in 35.3% of the patients (18 out of 51 patients) when compared to placebo (17.6%, 9 out of 52 patients) (*p* = 0.04) [71]. In a retrospective multicenter study, eighty-three patients with chronic pouchitis and CD of the pouch were treated with vedolizumab. Clinical response was obtained in 71.1% and clinical remission in 19.3% of the patients. A total of 54.1% of the 74 patients who underwent pouchoscopy reported endoscopic response and 17.6% of the patients had mucosal healing. The first episode of pouchitis within a year after IPAA was identified as the predictor for non-response to vedolizumab [72]. A case series of twenty patients treated with vedolizumab for antibiotic-dependent pouchitis and CARP demonstrated improvement in both PDAI and Oresland score at week 14 [73]. In a retrospective single-center study, twelve patients with CARP previously treated with thiopurines, infliximab or adalimumab (16.7%, 50.0% and 33.3%, respectively) received vedolizumab with clinical remission obtained in 81.8% of patients with steroid-free clinical remission in 63.6% and antibiotic-free clinical remission in 54.5% at week 14 [74]. Vedolizumab seems effective in biologic-experienced patients with chronic pouchitis. A clinical study that included 49 patients with CARP, out of which 31 (63%) were previously treated with a first line anti-TNF-α, showed clinical response in 17 (34%) and clinical remission in 3 (6%) patients at week 10. Long-term follow-up at week 52 revealed clinical response in 22 (44%) patients, clinical remission in 12 (24%) patients, endoscopic response in 15 (60%) and endoscopic remission in 5 (20%) cases [75]. A decrease in PDAI clinical subscore, a decrease in fecal calprotectin levels and an improvement in histologic inflammation were also obtained with vedolizumab for the treatment of CARP in a case series of thirteen patients [76]. In a single-center cohort of 19 patients, out of which 10 (53%) were previously treated with anti-TNF-α agents for CARP, vedolizumab showed both clinical and endoscopic improvement in 14 (74%) patients at three months [77]. In 2022, the European Commission approved intravenous vedolizumab for the treatment of adult patients with moderately to severely active chronic pouchitis who had undergone total proctocolectomy with IPAA for UC and had either an inadequate response with or lost response to antibiotics. Vedolizumab is the first drug approved by the European Medicine Agency for the treatment of active chronic pouchitis [78].

#### 5.4.3. Anti-Interleukin (IL) 12/23

Ustekinumab was studied on twenty-four patients with chronic pouchitis. Twelve patients (50%) had clinical response and the PDAI endoscopic subscore decreased in all thirteen patients who underwent pouchoscopy after ustekinumab treatment [79]. Another prospective, multicenter open-label study for patients with CARP evaluated long-term ustekinumab therapy through week 48. Twenty-two patients were enrolled with steroid-free clinical remission rates of 27.3% and clinical response rates of 54.5% at week 16. Fourteen patients that continued ustekinumab through week 48 maintained favorable outcomes in both clinical and endoscopic PDAI subscores [80]. Ustekinumab optimization can help in obtaining clinical response in patients with inadequate response with standard dose. Both standard and optimized doses of ustekinumab were evaluated in a retrospective study of 46 patients with CARP. All patients were biologically experienced with prior anti-TNF-α, while 24 (52.2%) had prior vedolizumab. Thirty-seven patients (80.6%) had clinical response at 8 to 16 weeks after ustekinumab initiation and twenty-three (50%) patients underwent ustekinumab dose intensification every 6 weeks or every 8 weeks after an approximate period of 7 months, with clinical response achieved in 63.6% of patients [81]. A multicenter cohort study that assessed the efficacy of ustekinumab in inflammatory disorders of the pouch demonstrated clinical response in five of six patients with chronic pouchitis. Three out of five responders successfully stopped all antibiotics during the six months after ustekinumab initiation. However, none of the six patients achieved clinical remission [82].

#### 5.4.4. Small Molecules

A retrospective single-center study identified eight patients treated with tofacitinib for chronic pouchitis. Patients were previously treated with antibiotics, immunomodulators and biologics (anti-TNF-α, vedolizumab and ustekinumab). Only one patient had improvement in clinical symptoms and two patients required pouch excision [83]. Tofacitinib was effective in the induction of clinical remission in a multicenter open-label induction with randomized withdrawal trial. In this trial, 33 patients with chronic pouchitis received tofacitinib 10 mg twice daily with clinical and endoscopic response rates of 55% and 58%, respectively, at week eight. Eleven patients continued tofacitinib through week eight with a clinical response of 36% and an overall response rate, including an extended induction of 67% [84]. Another retrospective multicenter study included fifteen patients with CARP treated with Janus kinase (JAK) inhibitors and sphingosine-1 phosphate (S1P) receptor modulators. Eight patients were treated with tofacitinib while one discontinued treatment after 8 months due to loss of response; 50% of patients who continued tofacitinib for twelve months achieved steroid- and antibiotic-free clinical response and 16.7% of patients achieved steroid- and antibiotic-free clinical remission. Endoscopic response and endoscopic remission were seen in 50% of cases each. Of the six patients treated with upadacitinib, steroid- and antibiotic-free clinical response was achieved in 33.3% of cases and steroid- and antibiotic-free clinical remission in 16.7% of cases at three months. Two patients were treated with ozanimod, of which one patient achieved steroid- and antibiotic-free clinical response at three months [85]. A small case series of six patients with CARP and CD of the pouch treated with upadacitinib for at least six weeks showed no clinical and endoscopic improvement with the treatment [86].

## 6. Discussion and Future Directions

Pouchitis in patients with total proctocolectomy and IPAA for UC is frequent and a significant number of patients develop phenotypes that require multiple courses of antibiotics, long-term antibiotic use or do not respond to antibiotics and need the use of immunosuppressive treatments. Available data regarding therapeutic options in patients with pouchitis are scarce. Usually, these patients are excluded from randomized controlled trials with advanced therapies, and at the moment, therapeutic decisions are mostly empirical, based on reported case series or small trials. Larger randomized controlled trials are needed. Recently AGA formulated a clinical practice guideline on the management of pouchitis, but most of the recommendations have low to very low certainty of evidence [43]. The International Ileal Pouch Consortium initiative formulated a consensus guideline for the treatment of pouchitis and other inflammatory pouch disorders with most consensus statements having poor reference standards and a lower quality of evidence [32]. More randomized controlled trials to assess the efficacy of different molecules with a focus on biologics and small molecules are needed. Treatment targets in patients with pouchitis are not clearly established, with various treatment outcomes defined in different studies. Similar to IBD, concrete treatment options as well as concrete time points to achieve these outcomes are also needed for the management of pouchitis [87,88]. The development of adequate measurements for therapeutic response could also be useful. There are no validated scores in diagnosis and monitoring the disease course, and the available scores that are used in practice have a poor correlation between clinical symptoms and endoscopic activity [37,89,90]. Biomarkers such as fecal calprotectin, fecal lactoferrin or C-reactive protein should be further tested, and cut-off values should be established as noninvasive methods of assessing disease activity [38,91,92]. Classification of disease phenotypes should also be better defined for a targeted therapeutic approach [93]. For now, the main recommendation for the initiation of advanced therapies is represented by antibiotic-refractory disease. CARP is the main phenotype that was included in available studies with biologics and small molecules. Treatment options for recurrent antibiotic-dependent disease still include repeated antibiotic regimens or cycling between antibiotics or the long-term use of a less-effective dose [43]. Taking into consideration the long-term burden of antibiotic use, further studies should be designed to include this phenotype and future initiatives should suggest an earlier initiation of biologics or small molecules for this subgroup of patients. No data for novel molecules that include anti-IL-23 agents are available, and there are very few reported cases involving treatment with selective JAK inhibitors or S1P receptor modulators. However, for chronic pouchitis, either CARP or antibiotic-dependent, same immunosuppressive agents for the treatment of CD or UC can be used [43].

Further, we propose an algorithm for the management of pouchitis in clinical practice. For patients with a clinical suspicion of pouchitis (loose stools, increased frequency, abdominal pain, abdominal cramps, urgency), coproculture should first rule out infections, especially *Clostridium difficile* infection. Endoscopy should further be performed to discriminate between pouchitis and other conditions. A pouch with diffuse erythema, ulcerations, erosions and the loss of vascular pattern is diagnostic for pouchitis. In some cases, pre-pouch ileal involvement can include similar endoscopic aspects as the pouch body, which should not be misdiagnosed as the CD of the pouch in the absence of other more specific features (e.g., distal ileal ulcerations or fistulas). For the first episode of pouchitis, a four-week course of ciprofloxacin 1 g/day can be used. In case of recurrence, a four-week course of metronidazole 20 mg/kg/day or a combination with ciprofloxacin and metronidazole could also be appropriate. Vancomycin and rifaximin can be of benefit for some patients. From our clinical experience, the use of probiotics for both the prevention and treatment of pouchitis has shown low efficacy. For CARP and recurrent pouchitis, we suggest the initiation of advanced immunosuppressive therapies that include biologics or small molecules.

Recently, progress in the therapeutic management of IBD has drawn attention to the benefit of using somatic-cell-therapy medicinal products that comprise immune and stem-cell therapies. The purpose of these treatments is to replace damaged cells, induce mucosal tissue healing and modulate the local immune response. Cell-based therapies using regulatory T (Treg) cells and tolerogenic dendritic cells can restore altered mucosal integrity by modulating T-cell activity and reduce inflammation. Mesenchymal stromal cells have immunomodulating properties, express markers such as CD 90, CD 105 and CD 73, and have the capacity to differentiate into different cell types like adipocytes or osteoblasts. For the treatment of refractory UC, allogenic-umbilical-cord mesenchymal cells were safe and helped in clinical improvement [94]. In CD, therapy with adult allogenic-expanded adipose-derived mesenchymal stem cells (darvastrocel) has been approved for the treatment of perianal fistulizing refractory CD [95,96,97]. However, these therapies have not been studied for chronic pouchitis. Considering that among all of the immune and stem-cell therapies, mesenchymal-stem-cell therapy is the only therapy with the most robust data on efficacy for fistulizing perianal CD and that idiopathic pouchitis is characterized by an inflammatory phenotype, at the moment, there is no sufficient evidence that can support the benefit of these treatments in chronic idiopathic pouchitis. Further studies are needed to assess the benefit of these therapies both in the inflammatory phenotype of IBD and idiopathic pouchitis. Studies have shown that lifestyle changes like diet and physical activity may be of benefit for patients with IBD. Although there is evidence that highly processed food is associated with the development of IBD, during the disease course, proposed dietary interventions have shown conflicting data with regard to disease control [98,99]. IBD is associated with dysbiosis, and different strategies to restore gut microbiota have been intensively studied. Physical activity was associated with the modulation of the gut microbiota through several mechanisms: an increase in the gut levels of immunoglobulin A, a reduction in fecal bile acids, an increase in the production of short-chain fatty acids, and increased levels of 5-adenosine monophosphate-activated protein kinase. Also, physical activity can help to increase lean body mass and prevent sarcopenia that is associated with inflammatory burden in IBD [100,101]. However, there are insufficient data to support the beneficial role of physical activity in controlling IBD course [102]. There are no studies available to assess the role of diet or physical activity in patients with pouchitis.

## 7. Conclusions

Pouchitis is the most common inflammatory complication in patients with UC who underwent total proctocolectomy with IPAA. Current treatment options include antibiotics in infrequent episodes and advanced immunosuppressive treatments in chronic disease. Nevertheless, a significant number of patients require pouch excision secondary to refractory disease. Available data for the management of patients with chronic pouchitis are scarce. Larger randomized controlled trials for the management of chronic pouchitis should represent a topic of interest for further research.

## Figures and Tables

**Table 1:** PDAI score.

	Range
**I Clinical**	
**1. Stool frequency**	
Usual postoperative stool frequency	**0**
1–2 stools/day > postoperative usual	**1**
3 or more stools/day > usual postoperative usual	**2**
**2. Rectal bleeding**	
None or rare	**0**
Daily	**1**
**3. Fecal urgency and abdominal cramps**	
None	**0**
Occasional	**1**
Usual	**2**
**4. Fever > 37.8 °C**	
Absent	**0**
Present	**1**
**Maximal clinical subscore = 6**	
**II Endoscopic findings**	
1. Edema	**1**
2. Granularity	**1**
3. Friability	**1**
4. Loss of vascular pattern	**1**
5. Mucus exudates	**1**
6. Ulceration	**1**
**Maximal endoscopic subscore = 6**	
**III Histology—acute histological inflammation**	
**1. Polymorph infiltration**	
Mild	**1**
Moderate with crypt abscess	**2**
Severe with crypt abscess	**3**
**2. Ulceration per low-power field (average)**	
<25%	**1**
≥25% ≤50%	**2**
>50%	**3**
**Maximal histological subscore = 6** **Maximal total PDAI = 18**	
