# Correction: Meianu et al. Diagnosis and Medical Treatment of Acute and Chronic Idiopathic Pouchitis in Inflammatory Bowel Disease. *Medicina* 2024, *60*, 979

**DOI:** 10.3390/medicina60081208

**Published:** 2024-07-26

**Authors:** Corina Meianu, Tudor Stroie, Doina Istratescu, Carmen Monica Preda, Mihai Mircea Diculescu

**Affiliations:** 1 Gastroenterology Department, Fundeni Clinical Institute, 022328 Bucharest, Romania; 2 Faculty of Medicine, “Carol Davila” University of Medicine and Pharmacy, 050474 Bucharest, Romania

In the original publication [1], the following acknowledgement was not included:

**Acknowledgement:** Publication of this paper was supported by the University of Medicine and Pharmacy Carol Davila, through the institutional program “Publish not Perish”.

The authors state that the scientific conclusions are unaffected. This correction was approved by the Academic Editor. The original publication has also been updated.
